# Characteristic alterations of gut microbiota and metabolites in patients with perianal abscess: a multi-omics analysis

**DOI:** 10.3389/fmicb.2025.1557972

**Published:** 2025-07-17

**Authors:** Huangfu Ma, Jingjing Fang, Mengjie Li, Yige Qin, Yanmei Wang, Seong-Gook Kang, Kunlun Huang, Tao Tong

**Affiliations:** ^1^Proctology Department, China-Japan Friendship Hospital, Beijing, China; ^2^Key Laboratory of Precision Nutrition and Food Qu ality, Key Laboratory of Functional Dairy, Ministry of Education; College of Food Science and Nutritional Engineering; China Agricultural University, Beijing, China; ^3^Department of Food Engineering and Solar Salt Research Center, Mokpo National University, Muangun, Republic of Korea; ^4^Key Laboratory of Safety Assessment of Genetically Modified Organism (Food Safety), Ministry of Agriculture, Beijing, China; ^5^Beijing Laboratory for Food Quality and Safety, Beijing, China

**Keywords:** perianal abscess, gut microbiota, 16S rRNA sequencing, gut metabolites, LC-MS/MS

## Abstract

**Background/objectives:**

Various studies have highlighted the important associations between perianal abscess (PA) and gut microbiota and related metabolites. Nevertheless, the establishment of causal relationships between these associations remains to be determined. This study aimed to provide direct evidence and a comprehensive understanding of gut microbiota disturbance in children with PA through combined 16S rRNA sequencing and untargeted metabolomic analysis.

**Methods:**

Thirty three children with PA and 16 healthy controls (HC) were continuously enrolled, and the demographic and clinical characteristics of the subjects were collected. First, 16S rRNA sequencing was used to evaluate differences in the gut microbiota between children with PA and the HC group, and 10 children with PA and 10 children in the HC group were randomly selected for non-targeted metabolomic analysis.

**Results:**

There were significant differences in the gut microbiota diversity and composition between children with PA and HC. Comprehensive analyses revealed an elevation of the genus *Enterococcus* and a depletion of the phylum *Firmicutes* as well as *Eubacterium_hallii_group*, *Faecalibacterium*, *Blautia*, and *Fusicatenibacter* at the genus level in children with PA. Non-targeted metabolomics detected 1168 different metabolites between children with PA and HC. Functional analysis of the gut microbiota and KEGG pathway enrichment analysis of differential metabolites between the PA and HC groups corroborated that the pathways of phenylalanine, tyrosine and tryptophan biosynthesis, valine, leucine and isoleucine biosynthesis, and pantothenate and CoA biosynthesis were down-regulated in children with PA, while the pathways of ubiquinone and other terpenoid-quinone biosynthesis, tyrosine metabolism, and tryptophan metabolism were up-regulated in children with PA. The correlation analysis highlighted meaningful associations between the altered microbiota and specific metabolic profiles, further underscoring the complex interplay between microbial composition and metabolic function in the pathogenesis of PA in children.

**Conclusions:**

This study provides new evidence of the pathogenesis of PA. There are significant differences in the gut microbiota, metabolites, and metabolic pathways between HC and children with PA, and the differences in metabolites are related to specific changes in bacterial abundance. These findings provide a foundation for future studies aimed at exploring targeted microbiome-based therapeutic strategies for managing PA in pediatric populations.

## 1 Introduction

Anal fistulas and perianal abscesses (PA) constitute prevalent anorectal pathologies in the pediatric population, including infants and children (Esposito et al., 2024). Clinically, these entities are intricately linked and frequently perceived as representing distinct phases within a singular disease process (Ding et al., 2021). The reported incidence of PA among infants ranges from 0.5 to 4.3%. The underlying etiology may encompass hormonal imbalances, anorectal crypt dysplasia, inflammatory bowel disease, immune deficiencies, diarrhea, and inadequate personal hygiene (Guner et al., 2023). In children, approximately two-thirds of these abscesses and fistulas are diagnosed prior to age two, predominantly affecting otherwise healthy individuals. Notably, there is a strong male preponderance, accounting for 92–100% of cases (Ezer et al., 2010).

The current management strategies for PA remain controversial, with a variety of approaches being employed. The traditional and most commonly adopted method involves prompt surgical drainage prior to spontaneous rupture of the abscess, aiming to prevent the dissemination of purulent material and the resultant loss of healthy tissue (Samuk et al., 2019). Nonetheless, surgical intervention is characterized by a substantial recurrence rate, spanning from 20 to 85%. In the case of fistulas, recurrence rates can reach as high as 68%, even after undergoing fistulotomy or fistulectomy (Festen and van Harten, 1998). The management of PA and anal fistulas in children remains a subject of debate, primarily due to the varying preferences of pediatric surgeons regarding the choice between conservative and surgical treatments, the utilization of antibiotics, and the timing of surgical intervention. Consequently, an optimal management protocol for PA and anal fistulas in pediatric patients has yet to be definitively established (Park, 2020).

Currently, associations between pediatric PA and gut microbiota are not comprehensively understood. While studies have investigated the potential involvement of aerobic and anaerobic organisms, including *Bacteroides fragilis*, *Peptostreptococcus*, *Prevotella*, *Fusobacterium*, *Porphyromonas*, various *Clostridium* species, *Staphylococcus aureus*, *Streptococcus*, and *Escherichia coli*, in the development of PA, the occurrence of these organisms has been linked to alterations in the gut microbiota (Brook, 2007; Wang et al., 2025; Yin et al., 2023). In adult populations, the presence of gut organisms within a PA has been identified as a robust predictor for the subsequent development of an anal fistula, significantly influencing the subsequent management approach (Tan et al., 2020). However, the precise role of gut-derived organisms remains unclear; they may contribute to the pathogenesis of anal fistulas or merely serve as a marker of a fistulous process in patients with anorectal abscesses (Sugrue et al., 2017). Elucidating the microbiota and metabolic profiles in affected pediatric patients could provide valuable insights into the underlying pathogenesis and potentially pave the way for novel therapeutic strategies.

To date, there have been limited studies examining the gut microbiota and associated metabolites in the context of pediatric PA. In our investigation, we utilized 16S rRNA sequencing and LC-MS/MS-based metabolomics to explore these relationships, with the objective of gaining a deeper understanding of how gut microbiota and metabolites influence the onset and progression of PA and fistulas in children. This study holds significant importance for preventing the colonization of potential pathogenic bacteria in pediatric patients with PA, as well as for advancing clinical treatment strategies and prognostic evaluations.

## 2 Materials and methods

### 2.1 Study subject recruitment

Participants newly diagnosed with PA from October to December 2022 were enrolled in this study. The inclusion criteria comprised a confirmed diagnosis of PA, an age range of 6 months to 3 years, consent to participate in the research, and comprehension of the study’s objectives. To enhance the precision of the findings and mitigate the influence of confounding variables, specific exclusion criteria were established: a history of severe cardiac, cerebral, hepatic, renal, intestinal, or psychiatric diseases, as well as recent antibiotic use within the past month. Meanwhile, 16 age and sex-matched healthy children were also were recruited as a control group. Ultimately, 33 patients with PA (PA group) and 16 healthy controls (HC group) were recruited from China-Japan Friendship Hospital. The research was approved by the ethics committee of China Agricultural University (CAUHR-20230901).

### 2.2 Sample collection

Fecal samples were collected from 16 healthy individuals and 33 patients with PA for subsequent experimental analysis. Specifically, stool specimens were obtained from all participants at the time of their enrollment, immediately after defecation, using sterile 10 mL centrifuge tubes. These fresh samples were promptly frozen in liquid nitrogen and stored at −80°C for preservation. Within 6 months of collection, the frozen samples were processed and dispatched to Shanghai Majorbio Bio-pharm Technology Co., Ltd. for comprehensive analysis, including 16S rRNA gene sequencing and untargeted metabolomic profiling.

### 2.3 Fecal DNA extraction and 16S rRNA gene sequencing

Fecal DNA was extracted using a fecal DNA isolation kit (Omega Bio-Tek, Georgi, United States). DNA samples were examined for concentration and purity using a NanoDrop 2000 spectrophotometer. The 16S rRNA gene segments (V3–V4) were amplified from extracted DNA using the primers 806R (5′-GGACTACHVGGGTWTCTAAT-3′) and 338F (5′-ACTCCTACGGGAGGCAGCAG-3′). The PCR conditions were: 30 s at 95°C, 30 s at 55°C, and 45 s at 72°C for 27 cycles. Paired-end sequencing of amplicons was performed using the Illumina MiSeq sequencing platform and PE300 chemistry at Majorbio Company.

### 2.4 Microbiota data analysis

The raw data were analyzed on the Majorbio Cloud platform.^1^ After demultiplexing, the sequences were combined with FLASH (version 1.2.11) and quality filtered with fastp (version 0.19.6). High-quality sequences were denoised using the DADA2 plugin in the QIIME (version 2020.2) pipeline. Amplicon sequence variants (ASVs) were the usual terms for DADA2 denoised sequences. The built-in QIIME Naive Bayes consensus classifier was used for ASV classification status assignment, while the SILVA 16S rRNA database (version 138) was employed for ASV categorization.

The α diversity at the ASV level was measured in QIIME. Principal coordinates analysis (PCoA) visualizations were created on the Majorbio Cloud platform, followed by Bray-Curtis distance-based analysis of similarity (ANOSIM). The differences between the bacterial taxa from the phylum to genus level were assessed via linear discriminant analysis (LDA) and LDA effect size (LEfSe) analysis, using thresholds of *p* < 0.05 and LDA score > 4. Due to the potential for high false-positive rates using LEfSe as described previously (Nearing et al., 2022), an analysis of the composition of microbiomes (ANCOM) was performed using the ANCOM 4.0.2 package in R to refine the identification of the bacterial taxa enriched across the sample groups. The W-statistic cutoffs from the ANCOM output (0.6, 0.7, 0.8, and 0.9) were used to identify taxa with significant differential abundance. A random forest model was built using the Majorbio Cloud platform to find biomarkers most likely to be related to PA status, and ROC analysis was performed on the random forest model. PICRUSt was employed to predict the function of the gut microbial communities, and an unpaired two-tailed Student’s *t*-test was used for statistical analysis (*p* < 0.05).

### 2.5 Fecal metabolomic analysis

Shanghai Majorbio Bio-pharm Technology Co., Ltd. performed untargeted metabolomic analysis of human fecal samples (*n* = 10, randomly selected). Metabolites were extracted using 400 μL methanol (4:1, v/v) solution with 0.02 mg/mL L-2-chlorophenylalanine as an internal standard. The mixture was allowed to settle at −10°C and treated by the High Throughput Tissue Crusher Wonbio −96°C at 50 Hz for 6 min, followed by ultrasound at 40 kHz for 30 min at 5°C. The samples were then placed at −20°C for 30 min to precipitate proteins. After centrifugation at 13,000 × g at 4°C for 15 min, the supernatant was carefully transferred to sample vials for liquid chromatography with tandem mass spectrometry (LC-MS/MS) analysis. The LC-MS/MS analysis was conducted on a Thermo UHPLC-Q Exactive HF-X system equipped with an ACQUITY HSS T3 column (100 mm × 2.1 mm i.d., 1.8 μm; Waters, United States). Mass spectrometric data were collected using a Thermo UHPLC-Q Exactive HF-X Mass Spectrometer equipped with an electrospray ionization (ESI) source operating in positive and negative modes.

The LC-MS raw data were converted into a common format using Progenesis QI software (Waters, Milford, United States). The data matrix obtained by searching the database was uploaded to the Majorbio cloud platform (see text footnote 1) for analysis. Subsequent to normalization based on total peak intensity, the processed data were uploaded and imported into the Ropls R package (version 1.6.2), where they underwent multivariate data analysis. This included partial least squares discriminant analysis (PLS-DA) to investigate alterations in metabolic profiles across different groups. The Student’s *t*-test was employed to determine the *p*-values between pairs of metabolite groups. Metabolites were screened based on a variable importance in projection (VIP) > 1.0 and *p* < 0.05. The Human Metabolome Database (HMDB) was utilized for annotating the identified differential metabolites. Differential metabolites between the HC and PA groups were imported into the Kyoto Encyclopedia of Genes and Genomes (KEGG) database for further analysis of their functions and metabolic pathways. Only pathways with *p* < 0.05 and impact value > 0.1 were considered as significantly altered. The Spearman correlations were analyzed via the Majorbio Cloud platform to clarify the relationships between the 62 differential metabolites involved in the 10 significantly altered metabolic pathways in the PA groups and the 15 differentially enriched gut bacteria at the genus level. The correlation coefficient diagram (|coefficient| > 0.7, *p* < 0.05) was plotted using Cytoscape (version 3.9.1).

### 2.6 Statistical analysis

All data were presented as means ± SEM. The unpaired two-tailed Student’s *t*-test was used in GraphPad Prism 9.5 (San Diego, California, United States) to assess the difference between the two groups, and *p* < 0.05 was considered statistically significant. The level of significance was denoted as follows: *p < 0.05, **p < 0.01, ***p < 0.001, and ****p < 0.0001.

## 3 Results

### 3.1 Clinical and demographic characteristics of the study participants

In total, 49 eligible cases in the discovery cohort including 33 treatment-naïve patients diagnosed as PA and 16 healthy individuals were included in this study. Clinical characteristics for each subject were provided in the Table 1. PA patients in our cohort tended to be younger, although we did not find statistical significance regarding these clinical parameters between PA and the healthy individuals (e.g., age, *p* = 0.31; the two-tailed *t*-test).

**TABLE 1:** Basic information about the study participants.

Group	HC	PA
Number	16	33
Age range	7 month–2 year	6 month–3 year
Average age	1.20 ± 0.14	1.02 ± 0.10

HC, healthy control; PA, perianal abscess.

### 3.2 Structural changes of gut microbiota in children with HC based on 16S rRNA data

**FIGURE 1:**
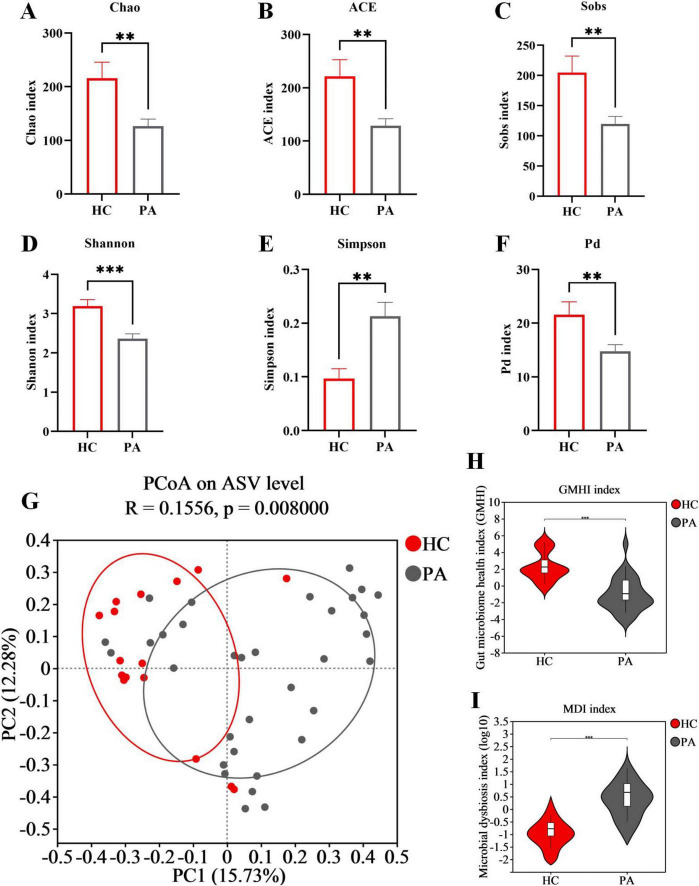
Structural changes of gut microbiota in children with HC based on 16S rRNA data. **(A)** Chao index. **(B)** ACE index. **(C)** Sobs index. **(D)** Shannon index. **(E)** Simpson index. **(F)** Pd index. **(G)** PCoA of the microbiota based on Bray-Curtis distance metrics (ANOSIM, R = 0.1556, *p* = 0.008). **(H)** Difference analysis of GMHI. **(I)** Difference analysis of MDI. Data are presented as mean ± SEM. PA group (*n* = 33) and HC group (*n* = 16). Statistically significant FDR correction **p < 0.01, ***p < 0.001, two-tailed Student’s *t*-test.

The α diversity represents the species evenness and richness within the microbiota, while β diversity represents the differences in microbial communities between different ecological samples. First, we analyzed the α diversity of the gut microbiota in the two groups. Our findings revealed that the community richness (assessed by the Chao, ACE, and Sobs indices, Figures 1A–C), community diversity (assessed by the Shannon and Simpson indices, Figures 1D,E), and phylogenetic diversity (assessed by the Pd index, Figure 1F) of the gut microbiota were significantly decreased in the PA group compared to the HC group. Next, we evaluated the β diversity of the two groups. PCoA based on the Bray-Curtis distance was used to evaluate the β diversity of the samples. As shown in the Figure 1G, the distance between the samples was represented by the two principal coordinates (PC1 and PC2). There was a significant separation between the gut microbiota of HC and PA children, indicating a significant change in the structure of the gut microbiota between healthy children and those with PA (*p* < 0.05). Through α and β diversity analysis, we showed that the structure of the gut microbiome was significantly different between healthy controls and patients with PA.

The gut microbiome health index (GMHI) was employed as a metric to assess the health status of the microbiota (Gupta et al., 2020). Extending this observation, we further discovered that the GMHI was decreased in children from the PA group compared to those in the HC group (Figure 1H). Additionally, the microbial dysbiosis index (MDI) was calculated to quantify the degree of microbial imbalance. The results indicated a significant increase in MDI in PA children compared to those in the HC group (Figure 1I). Collectively, these findings suggest that children with PA have a significant disruption and dysregulation of gut microbiota.

### 3.3 Alterations of gut microbiota composition in children with PA based on the 16S rRNA data

**FIGURE 2:**
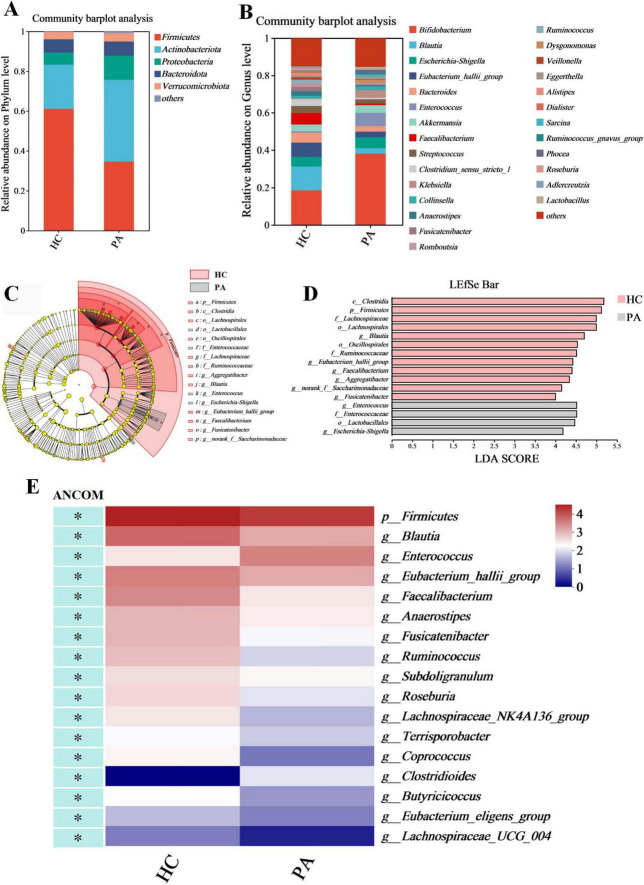
Identification of characteristic taxa with the most significant difference between the HC and PA groups. **(A)** Taxonomic proportions at the phylum level. **(B)** Taxonomic proportions at the genus level. **(C)** Cladograms generated by LEfSe indicate differences in the bacterial taxa between the HC and PA groups. **(D)** LDA scores indicating differentially abundant bacterial taxa between the HC and PA groups (LDA > 4). **(E)** A heatmap depicts the differential abundance of microbial taxa. Rows (microbial taxa at the phylum and genus level) and columns (samples) were ordered by hierarchical clustering. Differentially abundant taxa were determined by ANCOM (W > 0.7) and labeled with * in the heatmap. PA group (*n* = 33) and HC group (*n* = 16).

We examined the compositional differences in the gut microbiota at the phylum and genus levels between the two groups (Figures 2A,B). PA patients showed marked differences in composition compared to the healthy controls. At the phylum level, *Firmicutes* was the dominant phylum in the two groups (61.04% in HC and 34.69% in PA), followed by *Actinobacteriota* (22.30% in HC and 41.08% in PA), *Proteobacteria* (6.04% in HC and 12.14% in PA), *Bacteroidota* (6.67% in HC and 7.02% in PA), and *Verrucomicrobiota* (3.61% in HC and 4.17% in PA) (Figure 2B). Compared to the healthy children, PA children exhibited depletion of *Firmicutes*, and enrichment of *Actinobacteriota* and *Proteobacteria* relative to the levels. The PA group had a lower *Firmicutes*/*Bacteroidota* ratio than the HC group (9.14 in HC vs. 4.94 in PA).

At the genus level, the top 10 genera were *Bifidobacterium* (18.53% in HC and 38.03% in PA), *Blautia* (12.72% in HC and 2.91% in PA), *Escherichia-Shigella* (5.11% in HC and 5.90% in PA), *Eubacterium_hallii_group* (7.60% in HC and 3.01% in PA), *Bacteroides* (5.29% in HC and 2.97% in PA), *Enterococcus* (0.80% in HC and 7.11% in PA), *Akkermansia* (3.61% in HC and 4.17% in PA), *Faecalibacterium* (6.11% in HC and 0.79% in PA), *Streptococcus* (3.79% in HC and 2.40% in PA), and *Clostridium_sensu_stricto_1* (3.74% in HC and 0.91% in PA) (Figure 2B). The PA group showed significantly increased abundances of *Bifidobacteriaceae* and *Enterococcus* as well as reduced abundances of the genera *Blautia*, *Eubacterium_hallii_group*, *Bacteroides*, *Faecalibacterium*, and *Clostridium_sensu_stricto_1* compared to the HC group (Figure 2B).

LDA was integrated with LEfSe to identify the ASVs that were primarily responsible for the compositional differences observed in the gut microbial communities. The histogram of the LDA value distribution (LDA score > 4 and *p* < 0.05) showed that there were 16 differentially abundant taxa at different taxonomic levels, with 4 from the PA group and 12 from the HC group (Figures 2C,D; Supplementary Table S1). The class *Clostridia* had the largest LDA score (Figure 2D and Supplementary Table S1). The dominant taxa in the HC group were *Firmicutes* at the phylum level; *Clostridia* at the class level; *Oscillospirales* and *Lachnospirales* at the order level; *Ruminococcaceae* and *Lachnospiraceae* at the family level; and *Aggregatibacter*, *Eubacterium_hallii_group*, *Faecalibacterium*, *Blautia*, *norank_f__Saccharimonadaceae*, and *Fusicatenibacter* at the genus level. In contrast, the dominant taxa in the PA group were *Lactobacillales* at the order level; *Enterococcaceae* at the family level; *Enterococcus* and *Escherichia-Shigella* at the genus level.

Given the potential for high false-positive rates when using LEfSe (Tan et al., 2020), the ANCOM method was also employed to refine the identification of the bacterial taxa enriched across group comparisons. ANCOM (W > 0.7 and *p* < 0.05) confirmed that *Firmicutes* at the phylum level, as well as *Blautia*, *Eubacterium_hallii_group*, *Faecalibacterium*, *Anaerostipes*, *Fusicatenibacter*, *Ruminococcus*, *Subdoligranulum*, *Roseburia*, *Lachnospiraceae_NK4A136_group*, *Terrisporobacter*, *Coprococcus*, *Butyricicoccus*, *Eubacterium_eligens_group*, and *Lachnospiraceae_UCG_004* at the genus level were differentially enriched in the HC group, while *Enterococcus* and *Clostridioides* at the genus level (W > 0.7) were differentially enriched in the PA group (Figure 2E and Table 2). Collectively, these results suggested significantly altered gut microbiome composition in the children with PA.

**TABLE 2:** ANCOM analysis between HC and PA groups.

Taxa name	W	Detected_0.9	Detected_0.8	Detected_0.7	Detected_0.6	PA vs. HC
*p__Firmicutes*	198	False	False	True	True	Down
*g__Ruminococcus*	274	True	True	True	True	Down
*g__Blautia*	271	True	True	True	True	Down
*g__Clostridioides*	267	True	True	True	True	Up
*g__Fusicatenibacter*	264	True	True	True	True	Down
*g__Faecalibacterium*	262	True	True	True	True	Down
*g__Enterococcus*	261	True	True	True	True	Up
*g__Roseburia*	256	True	True	True	True	Down
*g__Butyricicoccus*	247	False	True	True	True	Down
*g__Subdoligranulum*	240	False	True	True	True	Down
*g__Anaerostipes*	235	False	True	True	True	Down
*g__Terrisporobacter*	225	False	True	True	True	Down
*g__Coprococcus*	223	False	False	True	True	Down
*g__Eubacterium_eligens_group*	219	False	False	True	True	Down
*g__Lachnospiraceae_UCG_004*	219	False	False	True	True	Down
*g__Lachnospiraceae_NK4A136_group*	213	False	False	True	True	Down
*g__Eubacterium_hallii_group*	211	False	False	True	True	Down
*g__Parasutterella*	195	False	False	False	True	Down
*g__Christensenellaceae_R_7_group*	183	False	False	False	True	Down
*g__UCG_005*	183	False	False	False	True	Down
*g__Lachnospiraceae_ND3007_group*	182	False	False	False	True	Down
*g__Family_XIII_UCG_001*	182	False	False	False	True	Down
*g__Haemophilus*	176	False	False	False	True	Down

HC, healthy control; PA, perianal abscess.

### 3.4 Random forest classification

**FIGURE 3:**
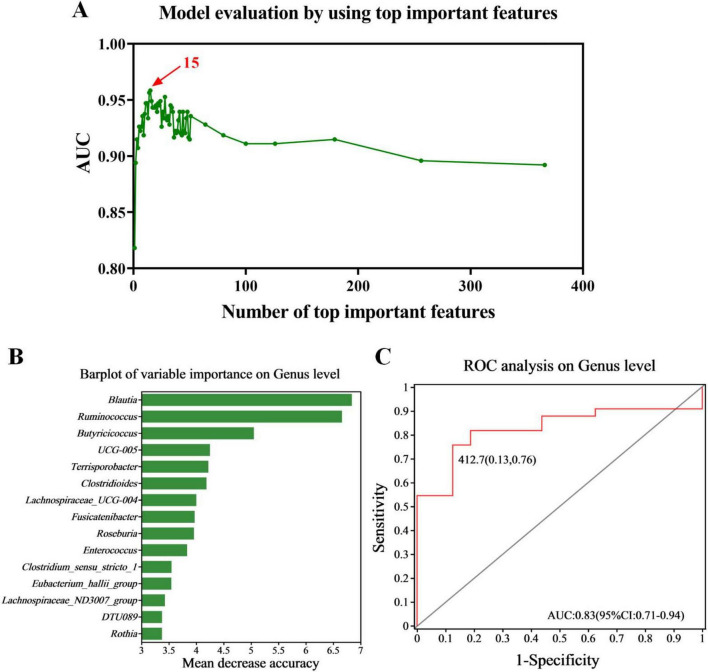
Diagnostic biomarker analysis using a random forest model. **(A)** Model evaluation by using top important features. **(B)** Barplot of variable importance at the genus level. **(C)** ROC curve of the 15 microbial genera model. PA group (*n* = 33) and HC group (*n* = 16).

We conducted random forest analysis on the gut microbiota at the genus level based on all 49 samples, to identify the key taxa that have a significant impact on the differences in gut microbiota between normal individuals and children with PA. We found that 15 genera (including *Blautia* and *Ruminococcus*) were important in distinguishing between the two groups (Figures 3A,B). Therefore, we constructed the best classifier model using the above 15 bacteria. The area under the curve (AUC) analysis result indicates that the above 15 bacteria can effectively distinguish children with PA from normal individuals with an overall AUC of 0.83 (95% CI: 0.71–0.94) (Figure 3C). Among the 15 genera, the genera *Blautia* and *Ruminococcus* were confirmed to have significantly more discriminatory power than others (Figure 3B), indicating that they were good diagnostic markers. In addition, *Blautia* showed the highest diagnostic value, which partly validated the results of LEfSe (Figure 2D) and ANCOM (Table 2).

### 3.5 Metabolomics analysis revealed aberrant metabolic patterns in children with PA

**FIGURE 4:**
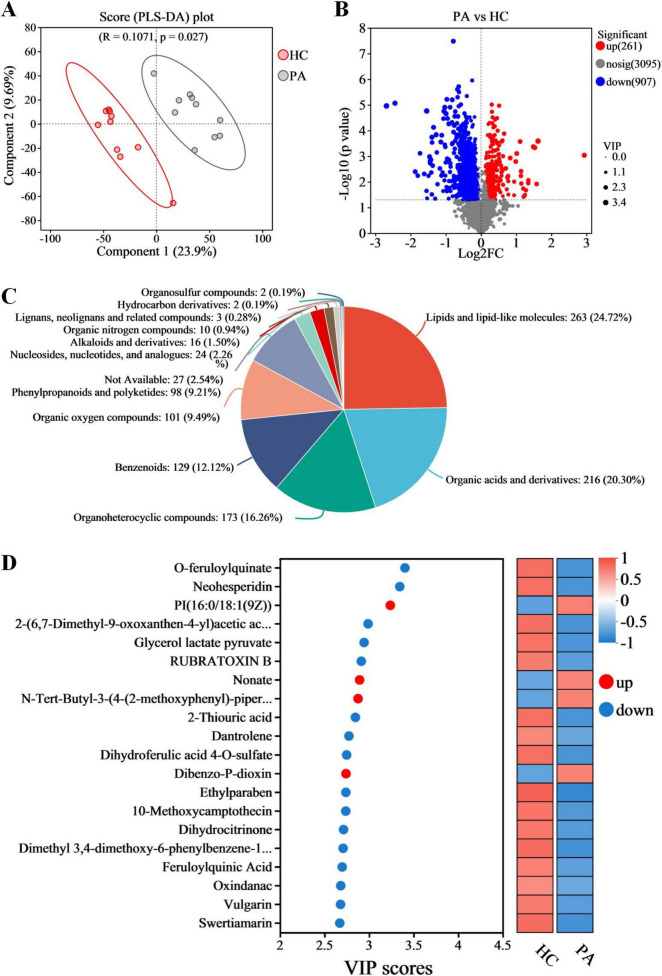
Metabolomic analysis reveals metabolic alterations in gut metabolic profiles in children with PA. **(A)** Partial least squares discriminant analysis (PLS-DA). **(B)** Volcano plots of the differential metabolites between HC and PA groups. **(C)** The proportion of differential gut metabolites categories. **(D)** Variable importance in projection (VIP) scores of the top 20 differential gut metabolites. *n* = 10.

In the present study, the non-targeted metabolomics analysis was carried out based on LC-MS technology to explore the differences in gut metabolites between children with PA and normal children. PLS-DA was also conducted to analyze the metabolic profiles based on class information. The PLS-DA model revealed significant disparities in metabolite composition between the PA and HC groups, suggesting unique gut metabolomic alterations in children with PA (Figure 4A). A total of 4263 metabolites were identified in the PA and HC groups. Based on VIP values > 1 (according to the PLS-DA model) and *p* < 0.05, 1168 differentially accumulated metabolites were identified, of which 261 metabolites were enriched and 907 metabolites were depleted (Figure 4B and Supplementary Table S2).

To gain a deeper understanding of the differences in gut metabolites between PA children and normal children and the molecular mechanisms involved, this study focused on the 1168 differential metabolites found between PA and HC groups. Metabolite annotation was conducted using the HMDB, revealing that 104 metabolites could not be annotated at the SuperClass level. The remaining 1064 metabolites were categorized as follows: lipids and lipid-like molecules (24.72%), organic acids and derivatives (20.30%), organoheterocyclic compounds (16.26%), benzenoids (12.12%), organic oxygen compounds (9.49%), phenylpropanoids and polyketides (9.21%), nucleosides, nucleotides, and analogs (2.26%), alkaloids and derivatives (1.50%), lignans, neolignans and related compounds (0.28%), hydrocarbon derivatives (0.19%), organosulfur compounds (0.19%), and an additional 2.54% that were not classifiable within these categories (Figure 4C).

The VIP score based on the PLS-DA model represents the potential of the metabolite as a biomarker and the top 20 metabolites (according to the VIP score) were considered important in the classification model (Figure 4D). Among these 20 metabolites, PI(16:0/18:1(9Z)), nonate, N-tert-butyl-3-(4-(2-methoxyphenyl)-piperazin-1-yl)-2-phenylpropanamide, and dibenzo-P-dioxin were significantly increased in the children with PA, while o-feruloylquinate, neohesperidin, 2-(6,7-dimethyl-9-oxoxanthen-4-yl) acetic acid, glycerol lactate pyruvate, rubratoxin B, 2-thiouric acid, dantrolene, dihydroferulic acid 4-O-sulfate, ethylparaben, 10-methoxycamptothecin, dihydrocitrinone, dimethyl 3,4-dimethoxy-6-phenylbenzene-1,2-dicarboxylate, feruloylquinic acid, oxindanac, vulgarin, and swertiamarin were decreased in the children with PA (Figure 4D).

### 3.6 Pathway enrichment analysis of metabolic pathways

**FIGURE 5:**
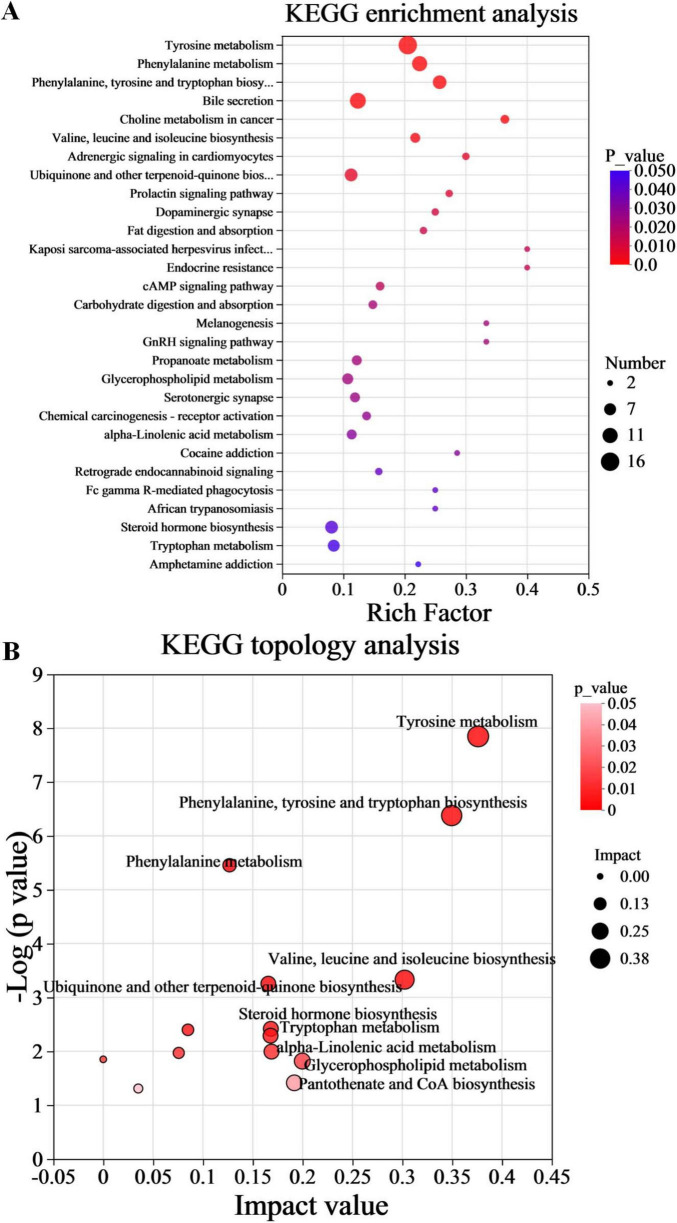
Functional enrichment analysis of gut differential metabolites. **(A)** Metabolic pathway enrichment of differential metabolites. **(B)** Topology analysis of metabolic pathways. *n* = 10.

A rigorous exploration into the significance of these metabolites was conducted through KEGG pathway enrichment analysis. The analysis of the 1168 differential metabolites between the PA and HC groups using KEGG enrichment identified 29 significantly altered pathways (Figure 5A, *p* < 0.05). Subsequently, to gain further insights into the biological pathways involved in the metabolism of the differentially expressed metabolites and their functional roles, the pathway enrichment, as well as topology analyses, were performed using MetaboAnalyst. Based on the identified metabolites and their concentration changes, 10 metabolic pathways emerged with a pathway impact value > 0.1, which served as the threshold for relevance. These 10 metabolic pathways, which were significantly altered in children with PA, were tyrosine metabolism, phenylalanine, tyrosine and tryptophan biosynthesis, valine, leucine and isoleucine biosynthesis, glycerophospholipid metabolism, pantothenate and CoA biosynthesis, alpha-linolenic acid metabolism, steroid hormone biosynthesis, tryptophan metabolism, ubiquinone and other terpenoid-quinone biosynthesis, and phenylalanine metabolism (Figure 5B, *p* < 0.05 and impact value > 0.1). The differential metabolites between PA and HC groups involved in the 10 metabolic pathways are presented in Table 3.

**TABLE 3:** The differential metabolites between PA and HC groups involved in the 10 metabolic pathways, with impact value > 0.1 and *p* < 0.05.

Pathway description	Impact value	*p*-value	Hits	Metabolite	PA vs. HC
Tyrosine metabolism	0.376106797	1.43346E-08	14	P-Coumaric Acid	Down
				Vanillylmandelic acid	Down
				Norepinephrine	Down
				2-(4-Hydroxyphenyl)ethanol	Down
				3,4-dihydroxyphenylacetic Acid	Down
				Tyrosol	Down
				L-Tyrosine	Down
				Gentisate Aldehyde	Down
				4-Hydroxyphenylacetaldehyde	Down
				Vanylglycol	Down
				Homogentisic acid	Down
				Gentisic acid	Down
				4-Hydroxyphenylpyruvic acid	Down
				Succinic acid	Down
Phenylalanine, tyrosine and tryptophan biosynthesis	0.349533716	4.24383E-07	10	Chorismate	Down
				Phenylpyruvic acid	Down
				2,4-Dihydroxybenzoic acid	Down
				Quinic acid	Down
				L-Tyrosine	Down
				5-Dehydroquinic acid	Down
				Shikimic acid	Down
				4-Hydroxyphenylpyruvic acid	Down
				Protocatechuic acid	Down
				3-Hydroxybenzoic acid	Down
Valine, leucine and isoleucine biosynthesis	0.302352941	0.000468321	5	(S)-2-Acetolactate	Down
				(S)-2-Aceto-2-hydroxybutanoic acid	Down
				2-Isopropylmalic acid	Down
				3-Isopropylmalic acid	Down
				2-Isopropyl-3-oxosuccinate	Down
Glycerophospholipid metabolism	0.199687695	0.015201736	8	PG(16:1(9Z)/16:1(9Z))	Up
				LysoPC(P-18:0/0:0)	Down
				PG(i-13:0/18:2(9Z,11Z))	Up
				PE(16:0/14:1(9Z))	Up
				PE(18:3(6Z,9Z,12Z)/P-18:1(9Z))	Down
				PC(14:0/P-18:1(9Z))	Down
				Hydroxyclomipramine	Down
				PA(10:0/i-12:0)	Down
Pantothenate and CoA biosynthesis	0.191680043	0.038561865	3	(S)-2-Acetolactate	Down
				Pantetheine 4’-phosphate	Down
				2-dehydropantoate	Down
Alpha-linolenic acid metabolism	0.168814433	0.010143705	4	Jasmonic acid	Down
				9-Oxo-nonanoic acid	Down
				PC(14:0/P-18:1(9Z))	Down
				13(S)-Hydroperoxylinolenic acid	Down
Steroid hormone biosynthesis	0.168152441	0.003831455	7	Androstenedione	Down
				Estrone	Up
				Dihydrocortisol	Up
				Estrone glucuronide	Up
				16b-Hydroxyestrone	Down
				3a,21-Dihydroxy-5b-pregnane-11,20-dione	Up
				21-Hydroxypregnenolone	Down
Tryptophan metabolism	0.167868039	0.005131416	7	5-Hydroxyindoleacetic acid	Down
				5-Methoxyindoleacetate	Down
				5-Hydroxyindoleacetate	Down
				Quinoline-4,8-diol	Down
				5-Hydroxy-L-tryptophan	Up
				2-Aminomuconic acid semialdehyde	Down
				L-Kynurenine	Up
Ubiquinone and other terpenoid-quinone biosynthesis	0.165608612	0.000555599	8	Chorismate	Down
				Isochorismate	Down
				P-Coumaric acid	Down
				(1R,6R)-6-hydroxy-2-succinylcyclohexa-2,4-diene-1-carboxylate	Down
				L-Tyrosine	Down
				Homogentisic acid	Down
				4-Hydroxyphenylpyruvic acid	Down
				Withanolide B	Up
Phenylalanine metabolism	0.126690482	3.51415E-06	9	Phenylpyruvic acid	Down
				M-Coumaric acid	Down
				N-Acetyl-L-phenylalanine	Down
				Phenylacetylglycine	Up
				L-Tyrosine	Down
				Phenylacetic acid	Down
				Hydrocinnamic acid	Down
				L-3-Phenyllactic acid	Down
				Succinic acid	Down

**FIGURE 6:**
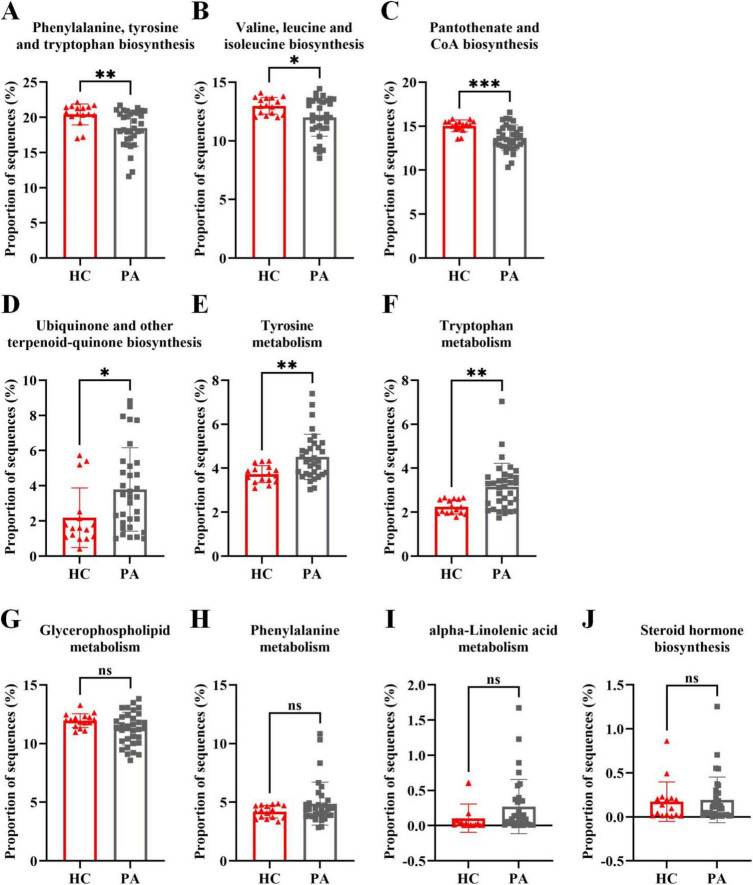
Metagenome prediction from PICRUSt between the HC and PA groups. **(A–C)** The pathways of phenylalanine, tyrosine and tryptophan biosynthesis, valine, leucine and isoleucine biosynthesis, and pantothenate and CoA biosynthesis were decreased in children with PA. **(D–F)** The pathways of ubiquinone and other terpenoid-quinone biosynthesis, tyrosine metabolism, and tryptophan metabolism were increased in children with PA. **(G–J)** The pathways of glycerophospholipid metabolism, phenylalanine metabolism, alpha-linolenic acid metabolism, and steroid hormone biosynthesis were not significantly altered in children with PA. The bars indicate mean ± SEM values. The data were statistically analyzed using the unpaired two-tailed Student’s *t*-test. Significance was set at ns, not significant (*p* > 0.05), *p < 0.05, **p < 0.01, and ***p < 0.001. PA group (*n* = 33) and HC group (*n* = 16).

Furthermore, the findings from the metabolomic analysis were substantiated and corroborated by the predicted functional analysis of the gut microbiota. To gain insights into the direct relationship between intestinal microbial changes and the metabolic profile changes in children with PA and normal children, PICRUSt was utilized to predict the potential functional pathways that could be affected by the gut microbiota. As anticipated from the microbiome data, the pathways of phenylalanine, tyrosine and tryptophan biosynthesis, valine, leucine and isoleucine biosynthesis, and pantothenate and CoA biosynthesis were decreased in children with PA (Figures 6A–C), while the pathways of ubiquinone and other terpenoid-quinone biosynthesis, tyrosine metabolism, and tryptophan metabolism were increased in children with PA (Figures 6D–F). Collectively, these results demonstrated that the metabolomic analyses aligned with several key observations from the microbiome studies, providing valuable insights into the differences in the gut microbiota and the metabolic profiles between children with PA and healthy controls.

### 3.7 Cross-correlation analysis between the microbiota and metabolites

**FIGURE 7:**
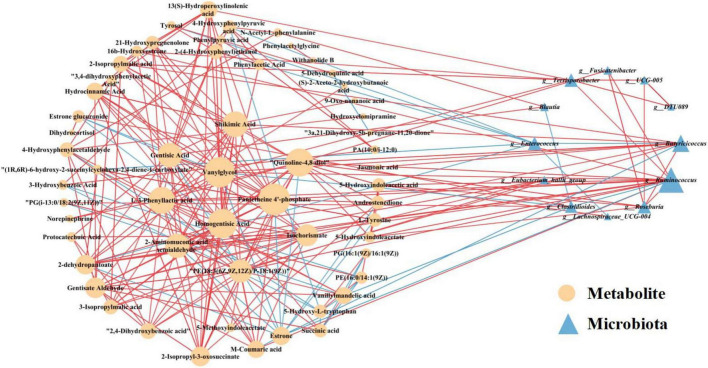
Correlation network analysis between the 62 differential metabolites involved in the 10 significantly altered metabolic pathways in the PA groups (as shown in Table 2) and the 15 differentially enriched gut bacterial genera (as shown in Figure 3B). Differential metabolites (depicted as orange circles) and differentially enriched gut bacterial genera (represented as blue triangles) are visualized as nodes. The size of each node represents its degree, reflecting the number of connections to other nodes within the network. The lines connecting these nodes represent statistically significant Spearman correlation coefficients (with *p* < 0.05 and |coefficient| > 0.7). Specifically, red links signify positive interactions between nodes, whereas blue links denote negative interactions.

To explore the functional relationship between altered gut microbiota and differentially accumulated fecal metabolites, we performed a correlation analysis based on Spearman correlation coefficients. We included the top 15 genera with statistical differences annotated at the genus level (identified by random forest model, shown in Figure 3B) and the 62 differentially accumulated metabolites involved in the 10 significantly altered metabolic pathways in the PA group (as shown in Table 2) for the analysis. This analysis was performed using the Spearman correlation method. In constructing the correlation network, only strong (|coefficient| > 0.7) and statistically significant (*p* < 0.05) correlations were considered for inclusion. The results indicated that the metabolites were correlated with the microbiota of PA patients and the network comprised 252 statistically significant correlations among the 62 differential metabolites and 15 differential gut bacterial genera (Figure 7), thereby providing a visual depiction of the intricate interdependencies between the gut microbiota and metabolites.

Several central nodes emerged in the network, forming numerous connections and thus likely serving as hubs of physiologically significant interactions. Based on the node degree, the top 2 gut microbial taxa identified were *Ruminococcus* and *Butyricicoccus* in descending order (Figure 7). Notably, the genus *Ruminococcus* exhibited extensive connectivity within the network, demonstrating correlations with 17 nodes included in the correlation analysis. Furthermore, *Butyricicoccus* displayed significant and strong correlations with 10 nodes. Moreover, according to the node degree from high to low, pantetheine 4’-phosphate, vanylglycol, and homogentisic acid represented the top three differential metabolites, showing significant strong correlations with 22, 21, and 20 nodes, respectively (Figure 7). These findings suggest robust associations between altered gut microbiota and differentially accumulated fecal metabolites in children with PA, indicating the interactions and dynamic balances between gut microbial composition and gut metabolites.

## 4 Discussion

An imbalance in the gut microbiota and its associated metabolites may contribute to the emergence and progression of pediatric PA. However, our understanding of the role of the gut microbiota in the pathogenesis of PAs remains limited. Consequently, there is a pressing need to conduct a thorough classification and functional characterization of the gut microbiota in individuals with PA. By employing 16S rRNA sequencing and metabolomic analysis, our study demonstrates the presence of gut microbiota dysregulation in children with PA. Specifically, certain intestinal bacterial genera and their corresponding functional alterations are intimately linked to the circulating metabolites associated with PA, highlighting a potential interaction between the host, intestinal microbiota, and metabolites that may be implicated in the pathogenesis of PA.

In terms of the gut microbiota, we found some differences in gut microbiota diversity, richness, and evenness in PA children compared with healthy children. There were significant differences between the α and β diversity analyses (Figure 1). And significant differences in bacterial abundances were observed at the phylum and genus levels (Figure 2). At the phylum level, our analysis revealed a significant depletion of the *Firmicutes* phylum and a decreased *Firmicutes*/*Bacteroidota* ratio in PA patients, in comparison to the control group (Figure 2). *Firmicutes* and *Bacteroidota* are the two primary bacterial phyla within the gut microbiota and are pivotal in maintaining intestinal homeostasis, as documented in previous literature (Yang et al., 2022). These two phyla have been implicated in a multitude of diseases, with alterations in the *Firmicutes*/*Bacteroidota* ratio serving as an initial indicator of gut dysbiosis (Abdallah et al., 2011; Lin et al., 2017). In accordance with these findings, studies have consistently reported a decrease in overall bacterial diversity among patients with inflammatory bowel disease, attributed to the loss of several butyrate-producing bacteria, notably including *Firmicutes* (Haac et al., 2019). Furthermore, a reduced *Firmicutes*/*Bacteroidota* ratio has been observed in other chronic autoimmune disorders, such as primary Sjögren’s syndrome, systemic lupus erythematosus, and systemic sclerosis (Moon et al., 2020; Yang et al., 2022).

At the genus level, our study demonstrated a significant increase in the abundance of *Enterococcus* in PA children (Figures 2C–E). This finding aligns with the research conducted by Yin et al., which reported a notable elevation in the relative abundance of *Enterococcus* in the surgery group compared to the control group (Yin et al., 2023). Additionally, *Enterococcus* has been frequently isolated as a common microorganism from cultures of patients with PA, as reported in previous studies (Chen et al., 2013; Liu et al., 2011). The heightened levels of *Enterococcus* may suggest a compromised gut environment conducive to the proliferation of pathogenic or opportunistic bacteria. Such conditions could exacerbate the inflammatory state and potentially perpetuate the cycle of infection and abscess formation in PA children.

In our study, comprehensive analyses utilizing both LEfSe and ANCOM demonstrated a significant depletion of the genera *Blautia*, *Faecalibacterium*, *Eubacterium_hallii_group*, and *Fusicatenibacter* within the pediatric asthma (PA) patient cohort, relative to control subjects (Figures 2C–E). These genera are recognized as pivotal bacteria in the human gut microbiome for their capacity to produce short-chain fatty acids (SCFAs), particularly butyric acid. Through rigorous random forest analysis, *Blautia* emerged as the most significant predictive biomarker for PA in the studied cohort (Figure 3B). *Blautia* has garnered considerable interest due to its established roles in alleviating inflammatory and metabolic diseases, as well as its antibacterial activity against specific microorganisms (Liu et al., 2021). The decreased presence of *Blautia* in PA children may suggest a diminished capacity for SCFA production, potentially contributing to weakened gut barrier function and deregulated immune responses. Furthermore, our findings revealed a significant decrease in the abundance of *Faecalibacterium* in PA children compared to controls (Figures 2C–E), aligning with the research conducted by Yin et al. (2023). *Faecalibacterium* is an abundant bacterial genus within the gut microbiome of healthy individuals and is capable of producing SCFAs, including butyrate (Ferreira-Halder et al., 2017). Various studies have extensively reported alterations in the abundance of *Faecalibacterium* in a range of intestinal and metabolic diseases in humans, such as colorectal cancer, Crohn’s disease, and ulcerative colitis (Benevides et al., 2017). Given its ubiquity and immunomodulatory properties, *Faecalibacterium* is often suggested as an indicator of, and an active contributor to, intestinal health and the maintenance of gut homeostasis (Miquel et al., 2013). Research indicates that assessing microbial abundance and identifying microbial types are crucial for elucidating the gut microbiota’s role in PA pathogenesis, as well as for enabling personalized treatment and preoperative assessment in PA patients (Yin et al., 2023). Consequently, the differentially abundant genera identified in this study (e.g., *Blautia*, *Faecalibacterium*, *Eubacterium_hallii_group*, and *Fusicatenibacter*) may hold significant clinical relevance for preoperative evaluation and personalized therapeutic strategy development in PA management, indicating that these genera merit further investigation.

Alterations in the microbiome frequently lead to modifications in metabolic profiles, which in turn influence the availability and diversity of nutrients and microbial metabolites (Melnik et al., 2017). Many studies have revealed that altered metabolite profiles are associated with various diseases (Sharon et al., 2014), but few studies have focused on PA children. In the present study, the metabolomic analysis indicated that PA children had unique metabolic characteristics compared to HC (Figure 4A), and a total of 1168 differentially accumulated metabolites were identified (Figure 4B). Through KEGG enrichment analysis and functional analysis of the gut microbiota, we found that the pathways of phenylalanine, tyrosine and tryptophan biosynthesis, valine, leucine and isoleucine biosynthesis, and pantothenate and CoA biosynthesis were decreased, while the pathways of ubiquinone and other terpenoid-quinone biosynthesis, tyrosine metabolism, and tryptophan metabolism were increased in children with PA (Figures 5, 6). Therefore, our study demonstrates that the occurrence and development of PA may be closely related to the synthesis and metabolism of tyrosine and tryptophan. Further research is needed to explore this possibility and develop targeted interventions for pediatric PA.

Some limitations of our research should be acknowledged. First, the total sample size was small. In studies of small samples, magnitude and sign errors are common because of the noisy nature of the data (Clayson et al., 2019). Thus, small sample sizes may undermine the replicability and generalizability of scientific research. Second, the influence of environmental, dietary, geographic, and other factors on the microbiota and metabolomic results should also be taken into account. We cannot ensure that our discoveries will apply to populations with diverse genetic backgrounds, lifestyles, and diets. It has been demonstrated that gut microbiota composition differs by country, suggesting that eco-geographic dietary variations shape the gut microbiota (De Filippo et al., 2010). Therefore, the results of the gut microbiota research should be interpreted carefully according to the ethnic, age, dietary, nutritional, geographic, gender, and socioeconomic status of the study population before applying those data to other groups of children (Tokuhara, 2021). Third, no comparison was made between PA children and other anorectal diseases. Finally, the observed results were not validated in animal models to better define the functional role of the identified microbiota and metabolites.

Understanding the gut microbiota composition in PA children opens new possibilities for therapeutic strategies aimed at restoring a healthy microbial balance. Interventions such as probiotics, prebiotics, dietary modifications, and potentially fecal microbiota transplantation could be explored to replenish beneficial SCFA-producing bacteria and restore gut health. Future research should focus on longitudinal studies to clarify the causal relationships between gut microbiota alterations and PA. Additionally, investigating the functional consequences of these microbial changes through metabolomic and transcriptomic analyses will be essential for developing targeted therapies.

## 5 Conclusion

The gut microbiota and fecal metabolic phenotype in PA children were measured through 16S rRNA gene sequencing and LC-MS/MS methods. Our research concluded that PA children had not only a significantly different gut microbiota but also significantly different fecal metabolites. In addition, correlation analysis indicated that the changes in some gut microbes were correlated with changes in metabolites. In conclusion, PA not only disturbs the gut microbiota at the abundance level but also alters the host’s metabolic homeostasis. In general, regulated gut microbiota-related metabolites may serve as a new entry point for mechanistic research on PA and as a tool for early prediction, diagnosis, and treatment. These findings shed light on potential microbiome-related mechanisms underlying PA and suggest avenues for targeted therapeutic interventions.

## Data Availability

The original contributions presented in the study are publicly available. The 16S rRNAamplicon sequence data can be found at: https://www.ncbi.nlm.nih.gov/, accession number: PRJNA1282348.

## Footnotes

1
https://cloud.majorbio.com

## Supplementary material

Supplementary Table S2 The differential metabolites between PA and HC groups with impact value > 0.1 and *p* < 0.05.
