# Supplementary material for: Characteristic alterations of gut microbiota and metabolites in patients with perianal abscess: a multi-omics analysis
Source: Front Microbiol. 2025 Jul 17;16:1557972. doi: 10.3389/fmicb.2025.1557972 (PMC12312607; doi:10.3389/fmicb.2025.1557972)
Supplement: Supplementary file 1 [file Table_1.docx]

**Table S1.** Table of LDA discrimination results between HC and PA groups.

| **Taxa name** | **LDA value** | ***p* value** | **~~Group~~** | **PA vs HC** |
| --- | --- | --- | --- | --- |
| ***c__Clostridia*** | 5.18 | 0.000104 | ~~HC~~ | down |
| ***p__Firmicutes*** | 5.12 | 0.000598 | ~~HC~~ | down |
| ***f__Lachnospiraceae*** | 4.99 | 0.000208 | ~~HC~~ | down |
| ***o__Lachnospirales*** | 4.99 | 0.000208 | ~~HC~~ | down |
| ***g__Blautia*** | 4.70 | 0.000005 | ~~HC~~ | down |
| ***o__Oscillospirales*** | 4.53 | 0.003482 | ~~HC~~ | down |
| ***f__Ruminococcaceae*** | 4.51 | 0.000866 | ~~HC~~ | down |
| ***g__Eubacterium_hallii_group*** | 4.42 | 0.011397 | ~~HC~~ | down |
| ***g__Faecalibacterium*** | 4.40 | 0.001012 | ~~HC~~ | down |
| ***g__Aggregatibacter*** | 4.34 | 0.040164 | ~~HC~~ | down |
| ***g__norank_f__Saccharimonadaceae*** | 4.15 | 0.005633 | ~~HC~~ | down |
| ***g__Fusicatenibacter*** | 4.00 | 0.000355 | ~~HC~~ | down |
| ***g__Enterococcus*** | 4.52 | 0.003232 | ~~PA~~ | up |
| ***f__Enterococcaceae*** | 4.52 | 0.003232 | ~~PA~~ | up |
| ***o__Lactobacillales*** | 4.47 | 0.028094 | ~~PA~~ | up |
| ***g__Escherichia-Shigella*** | 4.18 | 0.034707 | ~~PA~~ | up |

HC: healthy control; PA: perianal abscess.
